# The paradox of canine conspecific coprophagy

**DOI:** 10.1002/vms3.92

**Published:** 2018-01-12

**Authors:** Benjamin L. Hart, Lynette A. Hart, Abigail P. Thigpen, Alisha Tran, Melissa J. Bain

**Affiliations:** ^1^ Department of Anatomy, Physiology and Cell Biology School of Veterinary Medicine University of California‐Davis Davis California 95616 USA; ^2^ Department of Population Health and Reproduction School of Veterinary Medicine University of California‐Davis Davis California 95616 USA; ^3^ Department of Medicine and Epidemiology School of Veterinary Medicine University of California‐Davis Davis California 95616 USA

**Keywords:** canine, coprophagy, dogs, faeces eating, stool eating

## Abstract

Canine conspecific coprophagy, the tendency or predisposition of some dogs to eat their own faeces or those of other dogs, seems paradoxical because dogs typically show an aversion to conspecific faeces. In an attempt to resolve this paradox, we set out to determine the factors associated with the occurrence of this behaviour and to evaluate the efficacy of 11 products marketed for treating coprophagy as well as behaviour modification procedures. Because a large sample of dogs was needed to address these issues, two web‐based surveys were utilized. One, intended to compare coprophagic dogs and non‐coprophagic dogs, yielded 1552 returns. The other, yielding 1475 usable returns, specifically recruited owners of coprophagic dogs to gather information about the characteristics of coprophagy and treatment success. The findings revealed that 16% of dogs sampled engaged in frequent conspecific coprophagy, defined as having been seen eating stools at least six times. No evidence was found relating the coprophagy to diet or the dog's age. Coprophagic dogs were as easily house trained as non‐coprophagic dogs, suggesting a normal aversion to faeces. Coprophagic dogs were more likely to be reported as greedy eaters than non‐coprophagic dogs. The reported success rate of the commercial products and behaviour modification approaches was close to zero, indicating that the behaviour is not readily changed. The coprophagy was overwhelmingly directed at fresh stools, defined as being no more than 2 days old. A hypothesis is offered that coprophagy reflects a tendency inherited from the ancestral wolf to keep the den area free of faecal‐borne intestinal parasites that might be deposited in the den resting area and would typically have parasite ova that are not initially infective, but could develop infective larvae after 2 days. An evolved parasite defence strategy to consume fresh faeces in the rest area would be adaptive.

## Introduction

A puzzling, but common, behaviour in some domestic dogs is a persistent tendency to consume their own faeces or those of other adult dogs. While there seems to be no clinically established abnormality associated with the behaviour, such as a gastrointestinal upset, nutritional deficiency or compulsive disorder, dog owners are often very disturbed by the behaviour. In fact, as of this writing, there were 11 commercial products specifically marketed for dealing with the problem: 21st Century Deterrence^®^; Coproban^®^; Deter^®^; Dis‐Taste^®^; For‐Bid^®^; Nasty Habit^®^; NaturVet Deterrent^®^; Potty Mouth^®^; S.E.P^®^; Stop Stool Eating^®^; Stop Tablets^®^.

A rather interesting paradox, presented by the occurrence of conspecific coprophagy, is that dogs seem to find conspecific faeces aversive and typically keep their ‘den’ areas clean by eliminating outside the house (Hart *et al*. 2006). This aversion to faeces is viewed as an innate behavioural adaptation inherited from wild wolf ancestors for avoiding exposure to faecal‐borne intestinal parasites and pathogens (Hart 1990, 2012). In nature, wolf and other canid faeces typically carry intestinal parasites as identified in scats (Bynum *et al*. 1977; Custer & Pencet 1981; Stancampo & Francisci 1993; Marquard‐Peterson 1997; Kloch & Bajer 2005).

There are no data‐based published studies dealing with the overall prevalence of conspecific coprophagy in domestic dogs or demographic factors, such as association with breed, gender, age, number of dogs in the household, diet or eating style. And, there are no data on the evaluation of efficacy of commercial products marketed specifically for the syndrome or the use of behaviour modification approaches to eliminate the problem long term.

One study reported that 28% of the dogs surveyed engaged in eating either herbivore or canine stools, but did not distinguish between the two behaviours (Boze 2008). A study of 14 coprophagic Labrador Retrievers found that punishing of attempts at eating stools with a citronella spray reduced the behaviour by about two‐thirds during the 3‐week trial, but the long‐term success was not reported (Wells 2003).

We addressed the topic of canine coprophagy with four objectives. One was to collect demographic data on the prevalence of conspecific stool eating by dogs in the general population and examine demographic factors such as gender, spaying or neutering, age, number of dogs in the household, type of food eaten, eating behaviour style and breed of the dog. The second objective was to look at the association of coprophagy with aversion, or absence of aversion, to conspecific faeces as indicated by ease or difficulty in house training. A third objective was to establish the characteristics of stool eating especially with regard to age of dog stools eaten. A fourth objective was to evaluate the therapeutic success of various behaviour modification approaches and the use of commercial products specifically marketed for treating stool eating. It was envisioned that learning about conspecific coprophagy in dogs might reveal some useful information in understanding and dealing with this problem behaviour.

Two contrasting testable hypotheses were considered. One is that coprophagic dogs exhibit an abnormal behaviour stemming from one or more contributing causes such as weak aversion to faeces, a dietary deficiency and association with a recognized compulsive behaviour. Depending on the possible cause, the predictions of this hypothesis were that: coprophagic dogs would be more difficult to housetrain than non‐coprophagic dogs, reflecting poor faeces aversion; coprophagic dogs would be fed a diet markedly different than that of non‐coprophagic dogs; and/or coprophagic dogs would be more likely than non‐coprophagic dogs to show one or more compulsive behaviours, such as tail chasing. Based on a presumed motivation for commercial production of food additives or pills for treating coprophagy, this hypothesis would also predict that one or more of the commercial products would be beneficial in some instances.

The second hypothesis was that coprophagic dogs may be exhibiting a variant of an innate behavioural predisposition, possibly stemming from wolf ancestors, that we hypothesize would have a tendency to keep the den resting area free of accumulating faeces left in the rest area by an injured or sick wolf. The behaviour would reduce the risk of parasitic infection from faeces just left alone. This hypothesis is covered more fully in the Discussion section where it is pointed out that infective forms of intestinal parasites become much more predominant after the faeces are over 2 days old. The predictions of this second hypothesis are: coprophagic dogs are as easily housetrained as non‐coprophagic dogs (reflecting normal aversion to faeces); and coprophagic dogs would tend to consume fresh faeces (no more than 2 days old) more than older faeces that in nature would contain infective parasite larvae. In contrast to the first hypothesis, another prediction is that this presumably innate predisposition would be very difficult to change by behaviour modification approaches or treatment with products specifically marketed for this syndrome.

## Methods

### Data collection

The types of information sought in this study required a large database, far beyond what one could obtain by interviewing dog owners. From past studies at this centre, and knowing that statistical analyses for the information sought would require responses from at least 1000 dog owners, two web‐based surveys were designed, carefully planned and pilot‐tested. Similar web‐based surveys have been used in a variety of data‐based behavioural and medical publications (McCobb *et al*. 2001; Gobar & Kass 2002; Janson & Wist 2004; Tynes *et al*. 2007; Sueda *et al*. 2008), and have been shown to provide data of a quality and validity comparable to traditional paper and pencil survey methods (Reips 2002; Rhodes *et al*. 2003; Gosling *et al*. 2004). The self‐administered surveys were intended to take 10–15 min of the respondents’ time.

Launched in 2010–11, the surveys were completed anonymously and voluntarily, with no personal identifiers, by interested dog owners recruited on dog listservs. As with other published and anonymous web‐based surveys from this centre, there was no follow‐up contact with those responding to the survey, and only the anonymous data responses to the survey were viewed. Therefore, no human subject committee approval was needed for such data use.

The two surveys were launched 6 months apart (SurveyMonkey^®^). One was intended to estimate the prevalence of coprophagy and compare coprophagic dogs and non‐coprophagic dogs, and was labelled, ‘Dog Behavior: The Rest of the Story’. The title and introductory information did not mention stool eating, and the specific stool‐eating questions were imbedded in a series of questions about the dog's diet, eating behaviour and behaviours not relevant to coprophagy (Appendix S1). The criterion for being coprophagic in this survey was that the dog had been seen eating stools at least six times. Table 1 provides a summary of question categories in this survey.

**Table 1:** Survey 1; Dog behavior, the rest of the story

Categories of Questions
Demographic data, such as number of dogs in household, sex, age, breed of dog
Yard space available to dogs
Ease of housetraining
Type of food given
Type of eater: finicky; greedy; normal
Dog's level of affection
Problem behaviors the dog has from list of 10
Howling at sirens
Eating non‐nutritional material other than grass or stools of dogs
Frequency of grass or plant eating
For stool eaters: frequency of eating stools: daily; weekly; monthly; yearly
For stool eaters: dog mostly eats only own stools; only stools of other dogs; both
For stool eaters: age of stools mostly eaten: 1–2 days; 2–4 days; >4 days

The second survey was labelled ‘Why Dogs Eat Their Stools’, and owners of coprophagic dogs were intentionally recruited so as to obtain detailed information on dogs that were well‐known by their owners to be frequently coprophagic (Appendix S2). Questions sought information on the age of stools that dogs consumed, the frequency of the behaviour and success in resolving the behaviour using products marketed for the problem and success with behaviour modification approaches. There was some overlap in questions between the two surveys, providing a cross‐check in reliability. Table 2 provides a summary of question categories in this survey. The criterion for keeping responses in the database was that the dog was seen eating stools more than 10 times and at least once a month to increase the likelihood that the responses regarding the details of stool eating were accurate.

**Table 2:** Survey 2; why dogs eat stools

Categories of Questions
Demographic data, such as number of dogs in household, sex, age, breed of dog
Ease of housetraining
Type of food given
Type of eater: finicky; greedy; normal
Dog's level of affection
Problem behaviors the dog has from list of 10
Frequency of grass or plant eating
Age that stool eating first noticed
Total times observed eating stools
Whether dog mostly eats only own stools; only stools of other dogs; both
Age of stools mostly eaten: 1–2 days; 2–4 days; >4 days
Frequency of eating stools: daily; weekly; monthly; yearly
Ways that you know a stool was eaten
Behavior modification treatments tried from a list of 7 and success of treatment
Commercial treatments tried from a list of 11 and success of treatment

### Statistical analyses

Chi‐square tests and Fisher's Exact tests for non‐parametric comparisons were used for pairwise comparisons between dogs specified as coprophagic, having been seen eating stools at least six times, and non‐coprophagic dogs, designated as never having been seen eating stools. The level of significance was set at *P* < 0.05, two‐tailed, and the Chi‐square value is given. In all cases, the Chi‐squared and Fisher tests produced qualitatively identical results; so, just the Chi‐square values are given. The logistic regression analysis was a stepwise logistic regression including only the variables that had a significance of *P* ≤ 0.01. This value was chosen to reduce the likelihood of a false positive of a variable. All analyses were run using SAS, version 9.4 (SAS Institute, Cary, North Carolina).

## Results

### Prevalence of conspecific coprophagia in dogs and demographics

The data for this section were from the survey, ‘Dog Behavior: The Rest of the Story’, where a total of 1552 useable responses were returned before the survey was closed and the data were gathered for analyses. The returns from the surveys came overwhelmingly from the United States (89.8%) and Canada (5.1%). For multi‐dog households, the respondents were told to choose the dog they knew best, or had known the longest, for answering questions; this was referred to as the specified dog. The specified dog could not be a mother with puppies, where some stool eating might be expected. Of the 1441 respondents answering the questions about conspecific stool eating, 76.9% (1108) reported never having seen their dog eating stools (referred to as non‐stool eaters or non‐coprophagic), while 16.0% (230) reported having seen their dogs eating stools ≥6 times (referred to as frequent stool eaters or coprophagic). Those reporting having seen their dogs eating stools 1–5 times (classified as neither coprophagic nor non‐coprophagic) were 7.1% (103). Accordingly, about 23% of the dogs sampled reportedly were seen eating stools at least one time. Depending upon how one categorizes a stool eater, that is, seen ≥6 times, or ≥1 time, the prevalence of stool eating among dogs represented by this survey ranged from 16 to 23%. Of the respondents with frequent stool eaters, 79.6% reported seeing their dogs eating stools greater than 10 times.

To make the contrast between coprophagic and non‐coprophagic dogs clear in the results presented below, unless otherwise noted, only dogs that were never seen eating stools were compared with dogs seen eating stools at least six times. An extensive table showing responses to each of the questions with all responses, and where dogs referred to as coprophagic are compared with non‐coprophagic dogs, is available in Appendix S3. In this survey, 82% of coprophagic dogs were described as consuming stools that were no more than 2 days old.

The occurrence of coprophagy was distributed among all four gender‐neuter groups and this measure did not distinguish between frequent stool eaters and non‐stool eaters. The distribution for neutered males, spayed females, intact males and intact females was 45.2%, 41.7%, 6.1%, and 7.0%, respectively, for stool eaters and 41.3%, 40.7%, 10.2%, and 7.8% for non‐stool eaters. Coprophagy does not seem to be a reflection of juvenile behaviour. Of coprophagic dogs 1.7% were less than 1 year of age and 75.1% were over 4 years of age, compared with non‐coprophagic dogs of which 3.2% were <1 year of age and 69.7% were over 4 years of age. Coprophagy does not seem to be related to age of separation from the dam. Of coprophagic dogs 59.1% were reported as being left with the dam for at least 7 weeks compared with 49.7% of non‐coprophagic dogs. Diet appears not to be related to coprophagy in that for 82.3% of frequent stool eaters, and 78.3% of non‐eaters, kibble was the main food.

An indication that coprophagy does not reflect a weak aversion to faeces is that 78% of dogs that were frequent stool eaters had been easily housetrained and remained well house trained, and a similar 82% of non‐stool eaters fell into this category of house training.

Coprophagy does not seem to be associated with the occurrence of compulsive‐like behaviours. Compulsive‐like behaviours were noted in 3.5% of frequent stool eaters and 2.9% of non‐eaters. The list of problem behaviours that could be noted, in addition to compulsive‐like behaviours by the responders, included separation anxiety, various types of aggressive behaviour, destructive behaviour and excessive barking, none of which were related to coprophagy.

Several variables were statistically associated with coprophagy, and Table 3 presents a stepwise logistic regression analysis of factors significantly related to coprophagy. The variable most highly associated with coprophagy was the reported eating style, with 51.1% of coprophagic dogs referred to as greedy eaters compared with just 28.2% of non‐coprophagic dogs.

**Table 3:** Stepwise logistic regression analyses of factors related to coprophagy

Factor	Parameter	Chi sq	*P*‐value
Greedy eating	0.86	27.90	<0.0001
Breed group	NA	20.79	0.0077
Multiple dogs in household	0.62	11.07	0.0005
Eating dirt	1.70	14.72	0.0001
Eating cat stools	0.51	10.84	0.001

See text for more details. The parameter measure for breed group evaluated nine different breed groups, each with a different value, so this is indicated as NA.

Breed identification was considered in two respects. One was with regard to breed group according to the American Kennel Club designation. In Table 3 of the logistic regression analysis, it can be seen that terriers and hounds are most likely to be coprophagic. With regard to specific breeds, the database, even with 1552 responses, could only provide limited information on individual breeds occurring frequently enough for comparisons. The specific breeds examined in this regard were those for which there were at least 15 dogs that met the criterion of being either coprophagic or non‐coprophagic. Shetland Sheepdogs (*N* = 27), with 41% being coprophagic, were overrepresented in comparison to 17% of other breeds (*P* = 0.003). On the other hand, with pooling of the three varieties of Poodles (Standard, Miniature, and Toy) (*N* = 29), none of the dogs was coprophagic (*P* = 0.006). Given that about 90% of the responses came from the United States, and the presumed differences in dog breeds between the United States and other countries, the information about breed groups or specific breeds is offered as relevant mostly to the United States.

The number of dogs in the household was important with dogs living in households with two or more dogs most likely to be coprophagic. While eating non‐nutritional substances was asked about in the survey, there was no question about the opportunity of the dog to eat the various non‐nutritional substances, such as horse or cattle stools. However, because dirt and cat stools would be frequently around, these were left in the logistic regression analysis. Having been reported as eating dirt and eating cat stools were positively associated with coprophagy.

### Specific characteristics of coprophagia

The data for this section were from the survey entitled, ‘Why Dogs Eat Their Stools’. There were 2561 returns before the survey was closed. Inclusion criterion were then applied, one being that the dog had to have been seen eating stools greater than 10 times. This criterion was posed in a question giving several options: 1–5 times, 6–10 times and greater than 10 times. A second criterion applied was that stool eating had to have been observed at least on a weekly basis. This criterion was posed in a question giving several options: daily, weekly, monthly, yearly and less than once a year. These rather demanding criteria were considered necessary to focus on dogs that were reliably coprophagic and where the behaviour was observed frequently. The survey with these criteria yielded 1475 returns, of which 62% ate stools daily and 38% weekly.

Coprophagic dogs from this survey were 30% neutered males, 42% spayed females, 9% intact males and 19% intact females. In this survey, 74% of the dogs had been housetrained easily, similar to the 78% of frequent stool eaters being easily housetrained in the first survey. With regard to eating style, in this survey, 52% were referred to as greedy eaters, compared with an almost identical 51% of frequent stool eaters in Survey 1 being referred to as greedy eaters.

The coprophagic dogs in this survey primarily consumed stools that were no more than 2 days old – referred to as fresh stools – with 85% identified as eating fresh stools. This corresponds to Survey 1 with 82% of frequent stool eaters having been seen eating stools no older than 2 days. The two surveys, taken together, confirm that coprophagic dogs overwhelmingly consume fresh stools.

An important finding in this survey is that the coprophagy appears not to have been altered by either behaviour modification and/or management techniques attempted by caregivers. In descending order of frequency of use, the various procedures were: chase away from stools (*n* = 1048); reward the successful command of ‘leave it alone’ (*n* = 424); lace stools with pepper (*n* = 295); and punish by electronic or sound‐emitting collar (*n* = 56). The reported success rate was 1–2% except for ‘leave it alone’ which was slightly higher at 4%.

The responses regarding the success of the 11 food additives or tablets specifically advertised for treatment of coprophagy are given in Table 4. While we have no information about the degree to which respondents followed instructions given with the products, coprophagy appeared not to be meaningfully altered by any of the products. The number of responders using these products ranged per product from 6 to 352. The reported rate of success ranged from 0 to 2%.

**Table 4:** Food additives and pills marketed for coprophagia

Name of product	Responses for product	Per cent reporting success
For‐Bid^®^	352	1
Deter^®^	238	1
Dis‐Taste^®^	154	1
Coproban^®^	58	2
S.E.P^®^	58	0
Stop Stool Eat^®^	27	0
Stop Tablets^®^	26	0
Potty Mouth^®^	24	0
NaturVet Deter^®^	20	0
Nasty Habit^®^	13	0
21st Century^®^	6	0

Responders were given a list of products and asked to say if the stool eating in their specified dog was resolved. Shown are the 11 products found, as of the writing of the paper. Some of these are dispensed through a veterinarian and some sold over the counter. The survey did not explore the degree to which the respondent closely followed directions on the label.

## Discussion

As indicated by the Internet attention given to this behaviour, canine conspecific coprophagy is, undeniably, an important concern for owners of companion dogs. One purpose of this study was to estimate the prevalence of conspecific coprophagy in the general population of dogs, as well as determine what environmental, biological, and management factors might differentiate coprophagic dogs from those that are not coprophagic. Another purpose was to explore the frequency of stool eating for those that are coprophagic, the age of stools eaten and the success of dog owners in using various behaviour modification techniques to eliminate this behaviour as well as their success with any of the 11 products marketed specifically for treating canine coprophagy. Finally, there was a goal to offer a hypothesis for the occurrence of canine conspecific coprophagy in a broad population of dogs.

One finding from the first web‐based survey with 1552 usable responses was that 16% of dogs in general are coprophagic, defined as having been observed eating stools at least six times. The occurrence of dogs seen eating dog stools at least once was 23%. Thus, depending on how one defines canine coprophagy, the occurrence is between 16 and 23%. In this paper, a coprophagic dog is defined as one having been seen eating dog stools at least six times, and a non‐coprophagic dog as one never having been seen eating dog stools.

In contrasting coprophagic with non‐coprophagic dogs, it was found that there was no difference with regard to distribution among sex or neuter categories, age, diet, ease of house training or association with a compulsive behaviour. Coprophagy could not be ascribed to a lack of normal mothering, because where information was available, coprophagic dogs were as likely to have been left with the dam for over 7 weeks as non‐coprophagic dogs.

Several factors did, however, distinguish between coprophagic and non‐coprophagic dogs in a significant manner with a significance value of *P* ≤ 0.01 (chosen to reduce the likelihood of false positives). Coprophagic dogs were much more likely to be described as greedy eaters, and were more likely to be found in multi‐dog households, where presumably there would be a greater concentration of stools. Eating dirt and cat stools were positively associated with coprophagy, as was breed group, with terriers and hounds being most coprophagic. Of individual breeds for which sufficient numbers were reported, Shetland Sheepdogs were overrepresented and Poodles (all varieties) underrepresented.

With regard to specific information on coprophagy from the second survey with 1475 responses meeting the criteria where the dogs were seen eating stools over 10 times and at least once a week, 85% were reported to eat stools no more than 2 days old (fresh stools). Similar results were found in the first survey, confirming that coprophagic dogs overwhelmingly consume fresh stools.

The success in eliminating the coprophagia with the various behavioural procedures ranged from only 1 to 4% . The survey did not request information on whether the behavioural techniques were directed from a canine behaviour specialist or were just tried on the respondents’ own initiative. The reported success rate for food additives or tablets marketed for coprophagy ranged from 0 to 2%. The survey did not get into the extent to which the respondent did or did not follow instructions that may have come with the product.

Two contrasting hypotheses were formulated for explaining coprophagy. One is that coprophagic dogs are exhibiting an abnormal behaviour stemming from one or more contributing causes. The second is that coprophagy is an expression of an adaptive behaviour inherited from ancestral wolves. None of the findings reviewed above supported the first hypothesis. Coprophagic dogs seemed to be as easily house trained as non‐coprophagic dogs, which we assume is an indication of aversion to faeces. Therefore, we do not ascribe coprophagy to an abnormal lack of aversion to faeces.

The perspective of the second hypothesis refers to an adaptive behavioural defence against parasites of wolves living in nature where faeces of injured or sick pack members might be deposited in the rest areas near the den. If wolves were to remove the faeces from rest areas where infective larvae from intestinal parasites would become more numerous over time, consumption is the only method available. For the most frequently reported intestinal parasites in wolf faeces, larvae from ova expelled in faeces that can directly transmit parasites do not develop into infective forms for at least 2 days. A list of the reported intestinal parasites found in scats of wolves, with development times in faeces (Bowman 2014), is shown in Table 5. For the parasites in the faeces, the ova passed in the shed faeces develop into infective larvae after a few days, depending upon parasite species and ambient temperatures. Leaving the faeces alone would allow the ova to hatch into infective larvae that could be picked up on the hair of wolves and groomed off, thus transmitting the parasites. If the faeces are consumed while fresh, however, within about 2 days, the larvae will not yet have developed into infective forms and the risk is presumably much less.

**Table 5:** Intestinal Parasites found in scats of wild wolves and other canids

Helminths (parasitic worms)
Cestodes (tapeworms)
Taenia spp., Echinococcus spp. Require ingestion by an intermediate host before infecting definitive host
Trematodes (flatworms or flukes)
Alaria spp. Require two intermediate hosts in water. Common in wolves, not dogs
Nematodes (roundworms)
Ancylostomidae and Uncinaria spp. Hook worms. Larvae develop into infective forms in the ova 2–9 days after being shed in the faeces. Infective larvae then develop in the environment (soil) and can then penetrate the skin of hosts.
Trichuris spp. Whipworms. Infective larvae develop in ova of shed faeces after 10–25 days
Toxocara spp. Ascarids. Infective larvae develop in ova in shed faeces in 2–4 weeks
Strongyloides spp. Pinworms or threadworms. Infective larvae hatch from ova in faeces in 2–3 days, and typically infect through the skin. Infections are usually mild.
Coccidea (single cell parasites)
Isospora spp. Oocysts develop into infective sporulated oocysts in faeces in 3‐5 days

Listed above is a classification of different genera of intestinal parasites found in dogs and in scats of wild wolves (Bynum *et al*. 1977; Custer & Pencet 1981; Stancampo & Francisci 1993; Marquard‐Peterson 1997; Kloch & Bajer 2005). In wild canids, several species are generally mentioned. The various parasite species of each type have the same basic life cycle. Note that the various parasite types require either an intermediate host before being infective, or require at least 2 days of development in the faeces, before being infective to the canid that is the definitive host. Life cycle information summarized from various resources (Bowman 2014).

There are other, less frequently occurring parasites found in canid faeces, as well as pathogenic bacteria from sick individuals, that can be immediately infective, so the consumption of fresh faeces can be considered ‘the lesser of two evils’ – leaving them alone or immediately consuming them.

This hypothesis that some domestic dogs could be displaying a wolf‐like coprophagy behaviour is a parallel to other wolf‐like behavioural predispositions seen in dogs. In fact the finding that being a ‘greedy eater’ is the strongest differentiating variable associated being a coprophagic dog would seem to support this wolf origin of coprophagy because one would expect greedy eating to be a common wolf characteristic.

We are aware of no study on wolves (or other canids) in nature that involves detailed observations of stool eating in the rest areas. Indeed, according to the perspective presented here, one would expect this behaviour not only to be infrequent, but to be carried out swiftly so that an observer could easily miss seeing it. However, a comment by noted wolf authority L. David Mech that ‘wolves do commonly practice coprophagy, at least in captivity’ (Harrington & Asa 2003), offers support for this perspective, which was further reinforced by a personal communication with Mech.

In the current environment, intestinal parasites of domestic dogs are commonly prevented and/or treated by an anthelmintic, which could result in relaxed natural selection for behaviours that would be related to avoidance of intestinal parasites. Consequently, one would expect some dogs to be vigilant in consuming stools, others to have completely lost this behaviour and others to be stool eaters on a sporadic basis. Additional variability in coprophagy would have come about in the selective breeding of dogs over centuries where one could expect differences in selection against this behaviour. Consistent with this perspective, we found an apparent under‐representation of coprophagy in Poodles and over‐representation in Shetland Sheepdogs. We are not aware of any publicized comments in breed development literature that may have played a role in the selection against coprophagy, but given the repulsive nature of the behaviour to humans, one would expect some breeders to avoid breeding dogs that are frequently coprophagic.

While the coprophagic syndrome seems to be medically harmless, it is very disturbing for many dog owners. One publication discussing this syndrome notes that some people find it so disgusting that the bond with their dog is irreparably damaged to the point where euthanasia is considered (McKeown *et al*. 1988).

There are several caveats that need to be mentioned in this study. One is with regard to the finding of no successful results in treating coprophagy with any of the commercial products. To the best of our knowledge, there have been no clinical trials with designated procedures for recruiting subjects to be treated and assuring that treatment guidelines were followed. Thus, there could be treatments that would be effective if instructions were followed. Secondly, the rule‐out of a compulsive disorder could be studied by careful observations on a sample of frequently coprophagic dogs and treatment with a psychotropic medication considered effective for some compulsive disorders. Thirdly, the explanation referring to a hypothetical parasite defence of wolf ancestors has no substantiating field observations and should be considered tentative.

## Source of funding

Supported by the Center for Companion Animal Health University of California, Davis (# 2009‐54‐F/M).

## Conflicts of interest

The research was conducted in the absence of any commercial or financial relationships that could be construed as a potential conflict of interest.

## Ethics statement

The authors confirm that the ethical policies of the journal, as noted on the journal's author guidelines page, have been adhered to. No ethical approval was required as no animals were used in this research and responses were provided anonymously.

## Contributions

BLH and LAH conceived and designed the study; BLH, APT, AT and MJB performed data collection and data analyses; BLH and LAH wrote the paper; LAH, MJB, APT and AT edited the paper.

## Supporting information


**Appendix S1.** Dog behavior: The rest of the story.

**Appendix S2.** Why dog eat their stools.

**Appendix S3.** Responses to survey, dog behaviour.

**Appendix S4.** Responses to why dogs eat their stools.
