# Supplementary material for: The paradox of canine conspecific coprophagy
Source: Vet Med Sci. 2018 Jan 12;4(2):106–14. doi: 10.1002/vms3.92 (PMC5980124; doi:10.1002/vms3.92)
Supplement: Supplementary file 1 — Appendix S1. Dog behavior: The rest of the story. [file VMS3-4-106-s001.pdf]

# Dog Behavior: The Rest of the Story

## 1. Dog Behavior: The Rest of the Story

Most dog caregivers have heard about how to train a dog to sit, stay and come, and many have heard about preventing aggressive behavior, separation anxiety and house soiling. But there is are several unanswered questions, such as why dogs eat strange things, why they howl at fire engines and if they "catch" human yawns. At the Companion Animal Behavior Program in the Veterinary School at the University of California at Davis, we are conducting a voluntary web-based survey of the primary caregivers of dogs to gather information about the rest of the story. If you are the primary caregiver of an adult dog, your cooperation in taking about 10 minutes to fill out this confidential survey will be appreciated by thousands of dog caregivers . Thank you very much for completing this survey.
If you have more than one dog, please fill out the survey for one dog that you know best or have had the longest.

You must be 18 years of age to take this survey.

## 2. Description of Your Household and Dog

In this section please answer questions about the dog you are responding about and your household

### * 1. Number of dogs in your household

- One
- Two
- Three
- Four
- More than four

### * 2. Please estimate the outdoor yard space that this dog or dogs, if you have more than one, have daily access to

- 10 square meters (about 90 sq feet), such as 3x3 m or 10x9 ft
- Over 10 sq m and up to 50 sq m (450 sq ft), such as 5x10 m or 45x10 ft
- Over 50 sq m and up to 200 sq m (2000 sq ft), such as 10x20 m or 40x50 ft
- Over 200 sq m and up to 500 sq m (4500 sq ft), such as 50x10 m or 20x25 m
- Over 500 sq m

### * 3. With regard to the above question, if you have more than one dog which dogs have access to the space indicated above

- All the dogs I have
- Just one dog, the one I am responding about
- Two of my dogs
- Three of my dogs
- Four of my dogs

### * 4. What is the sex of the one dog that you are responding about?

- Male-intact
- Male-neutered
- Female-intact
- Female-spayed

## * 5. What is the age of this dog?

- ◯ Less than 1 year
- ◯ 1-3 years
- ◯ 4-6 years
- ◯ Greater than 6 years

## * 6. At what age did you adopt this dog?

- ◯ Less than 2 months
- ◯ 2 to 4 months of age
- ◯ 4 to 6 months of age
- ◯ 6 months to 1 year of age
- ◯ 1-3 years of age
- ◯ 4-6 years of age
- ◯ Over 6 years of age

## * 7. What do you know about your dogs mothering?

- ◯ Orphaned from mother and litter mates before 2 weeks of age
- ◯ Away from mother and litter mates after 2 weeks but before 7 weeks of age
- ◯ With mother for greater than seven weeks
- ◯ I do not know

Other (please specify)

*** 8. What is the breed of this dog?**

- ◯ A MIXED BREED
- ◯ Basset
- ◯ Beagle
- ◯ Bichon frise
- ◯ Boxer
- ◯ Bulldog
- ◯ Chihuahua
- ◯ Cocker spaniel
- ◯ Dachshund
- ◯ Doberman pinscher
- ◯ English springer spaniel
- ◯ German shepherd
- ◯ German shorthair pointer
- ◯ Golden retriever
- ◯ Great dane
- ◯ Labrador retriever
- ◯ Maltese
- ◯ Miniature pinscher
- ◯ Other
- ◯ Pekinese
- ◯ Pomeranian
- ◯ Poodle-miniature
- ◯ Poodle-standard
- ◯ Poodle-toy
- ◯ Pug
- ◯ Rottweiller
- ◯ Shetland sheep dog
- ◯ Shih Tsu
- ◯ Siberian husky
- ◯ Welsh corgi
- ◯ West highland white terrier

- ◯ Yorkshire terrier

Other breed (please specify)

* 9. Please tell us the country where you and the dog live

- ◯ USA
- ◯ Canada
- ◯ Mexico
- ◯ Other Central or South American Country (specify below)
- ◯ UK
- ◯ Germany
- ◯ France
- ◯ Spain
- ◯ Other European Country (specify below)
- ◯ Japan
- ◯ Korea
- ◯ India
- ◯ China
- ◯ Other Asian country (specify below)

Other (please specify)

## 3. Questions About General Behavior

The questions below go into general aspects of behavior

### * 10. What response below best describes the degree to which the spcified dog is affectionate?

- ◯ Very affectionate
- ◯ Moderately affectionate
- ◯ Relatively non-affectionate

Other (please specify)

### * 11. Regarding house training, which of the answers best describes the ease of house training of this dog?

- ◯ Was difficult to house train, and as an adult is still not well trained
- ◯ Was difficult to house train, although the dog is now well house trained
- ◯ Was easy to house train and remains well house trained
- ◯ Was almost completely house trained from the start (very few "accidents")

Other (please specify)

### * 12. What type of food is the main food given this dog

- ◯ Kibble (dry)
- ◯ Canned or semi-moist food
- ◯ Raw food
- ◯ People food

Other (please specify)

### * 13. What response below best describes your dog's eating behavior

- ◯ Finicky eater
- ◯ Greedy eater, "wolfs down the food"
- ◯ Normal eater, neither finicky nor greedy

Other (please specify)

*** 14. What problem behaviors, other than eating dog stools, does your dog seem to have? Select all that apply.**

- [ ] Separation anxiety
- [ ] Aggression to family members
- [ ] Aggression to other adults
- [ ] Aggression to other dogs in family
- [ ] Aggression to other non-family dogs
- [ ] Destructive behavior
- [ ] Excessive excitement
- [ ] Tail chasing or other compulsive-like behavior
- [ ] Excessive barking
- [ ] None of the above

Other (please specify)

## 4. Interesting Things About Dog Behavior

This questions deas with interesting things that dogs do that are little understood, such as howling at sirens, catching human yawns and eating non-food items such as grass, dirt, stones and stools.

*** 15. Catching yawns. All dog owners have seen their dogs yawn. We are interested in understanding if you dog seems to catch your yawns or those of other family members, much as humans catch each others yawns. You may choose more than one response.**

- [ ] I have never seen my dog catch my yawns or those of others
- [ ] I recall a few times when my dog seemed to catch yawns
- [ ] My dog definitely catches my yawns or those of others, but not often
- [ ] My dog frequently catches my yawns or those of others
- [ ] My dog catches MY yawns, BUT NOT those of others
- [ ] My dog catches yawns of OTHERS, BUT NOT my yawns
- [ ] My dog so regularly catches yawns that I play with the dog by yawning

Other (please specify)

*** 16. Howling at sirens. This question deals with whether or not your dog howls at sirens, such as fire engines and the degree to which this howling is reliable or loud**

- [ ] Never howls at any noise
- [ ] Occasionally howls at a loud siren, but the howl is not loud
- [ ] Occasionally howls at a loud siren, but when it occurs it is loud
- [ ] Frequently howls at a loud siren, and the howl is fairly loud
- [ ] Almost alway howls at a loud siren, but can be quieted by command
- [ ] Almost alway howls at a loud siren, and cannot be quieted by command

Other (please specify)

*** 17. Eating non-nutritional material. Some dogs are attracted to eating small stones, dirt, sand or stools of cats, horses, cattle and other animals aside from dog stools (dog stools and plants are asked about in other questions). Please indicate which, if any, of the following you have seen your dog eat more than one time.**

- Dirt
- Small stones
- Sand
- Cat stools
- Horse stools
- Cattle stools

Other (please specify)

*** 18. Eating stools of their own or of other dogs. Although generally disgusting, eating their own stools of those of other adult dogs, is sometimes seen in adult dogs. Please answer this question the about frequency with which this dog eats stools of his or her own or of other dogs.**

- Never
- 1-5 times total
- 6-10 times total
- Greater than 10 times
- Never seen but I know that this dog eats stools of dogs

Other (please specify)

*** 19. If you have a multi-dog household how many of these dogs have you seen eating dog stools more than once?**

- Only one
- Two
- Three
- Four
- More than 4

*** 20. What types of dog stools does this dog eat?**

- ◯ Only eats stools of other dogs
- ◯ Only eats its own stool
- ◯ Eats either its own or other dog's stools, whichever is available

Other (please specify)

*** 21. If this dog does eat dog stools, what is your best estimate of the frequency in which the dog engages in this behavior.**

- ◯ Never eats stools
- ◯ 1 time per day or more
- ◯ Less than once a day, but at least 1 time per week
- ◯ Less than once a week, but at least 1 time per month
- ◯ Less than once a month, but at least 1 time per year
- ◯ Less than once a year

*** 22. Please indicate, to the best of your knowledge, the age of the dog stools that your dog eats. Select all that apply.**

- ☐ Fresh stools, no more than 1 day old
- ☐ Stools 1 to 2 days old
- ☐ Stools 2 to 4 days old
- ☐ Stools older than 4 days old
- ☐ I do not know

Other (please specify)

*** 23. Eating grass or other plants. Sometimes dogs eat grass or other plants. Please answer this question about how often you have seen this behavior**

- ◯ Never
- ◯ At least once but less than 6 times
- ◯ 6 to 10 times
- ◯ More than 10 times.

Other (please specify)

*** 24. If this dog does eat grass or other plants, what is your best estimate of the frequency in which the dog engages in this behavior.**

- Never
- 1 time per day or more
- Less than once a day, but at least 1 time per week
- Less than once a week, but at least 1 time per month
- Less than once a month, but at least 1 time per year
- Less than once a year

Other (please specify)

*** 25. Access to plants that could be eaten. Please answer this question about where your dog has regular access to grass or other plants and could eat them if the dog wanted to.**

- Plants in the yard as well as plants in the house
- Plants the dog has access to during walks
- Plants in the yard only; no house plants are available
- Plants in the house available as small grass "garden" for the dog
- Plants in the house available as as small grass "garden" meant for the cat(s)
- Plants in the house, but not a grass garden meant for dogs or cats
- No plans are available inside or outside to this dog

Other (please specify)

*** 26. Please answer this question about whether or not your dog regularly appears sick or ill prior to eating plants and/or regularly vomits within an hour after eating plans (if the dog does eat plants).**

- Not applicable because this dog does not eat plants
- Plant eating is so infrequent that I cannot answer this question
- The dog usually does seem to be sick before eating plants
- The dog usually does vomit after eating plants
- The dog just seems normal before and after eating plants

Other (please specify)

## 5. A Final Word

Thank you for completing this survey. Our intention is to use the information, collected anonymously, to learn more about the "rest of the story" with regard to dog behavior.

**27. Feel free to briefly tell us about other interesting behaviors of dogs that we should include in a future survey.**
