# Supplementary material for: The paradox of canine conspecific coprophagy
Source: Vet Med Sci. 2018 Jan 12;4(2):106–14. doi: 10.1002/vms3.92 (PMC5980124; doi:10.1002/vms3.92)
Supplement: Supplementary file 2 — Appendix S2. Why dog eat their stools. [file VMS3-4-106-s002.pdf]

Why Dogs Eat Their Stools

1. Introduction: Why Dogs Eat Stools. UC Davis School of Veterinary Medicine

About the most perplexing behavior of some dogs is their habit of eating their own stools or stools of other dogs. As disgusting as this behavior is, we, as the caregivers, must deal with it. At the Companion Animal Behavior Program in the Veterinary School at the University of California at Davis, we are conducting a voluntary survey of the primary caregivers of dogs that eat stools of their own or other dogs, to develop ways to prevent and treat this behavior. If your dog engages in this behavior, your cooperation in taking about 10 minutes to fill out this confidential survey will be appreciated by the thousands of dog caregivers that are frustrated by their dog's stool-eating behavior. Thank you very much for completing this survey.

IF YOU HAVE MORE THAN ONE DOG THAT FITS THIS CATEGORY:
Fill out the survey for one dog. You will then have to close out your browse and re-enter, or clean out your "cache". We understand that this may be a challenge to do, so please do not feel obligated to do so.

You must be 18 years of age to take this survey.

2. Description of Your Household and Dog

Where relevant, please answer the questions about the dog that eats stools. If a multi-dog household, answer questions for the dog that most frequently eats stools

* 1. Number of dogs in your household

- ◯ One
- ◯ Two
- ◯ Three
- ◯ Four
- ◯ More than four

* 2. If you have a multi-dog household how many of these dogs have you seen eating dog stools?

- ◯ Only one
- ◯ Two
- ◯ Three
- ◯ Four
- ◯ More than 4
- ◯ None
- ◯ None...they only eat cat, horse, or other animal's stools

3. Description of Household and Dog Continued

Why Dogs Eat Their Stools

Reminder...the questions below are only regarding eating DOG STOOLS, not other animals' stools.

*** 3. What is the sex of the one stool-eating dog that you are responding about?**

- Male-intact
- Male-neutered
- Female-intact
- Female-spayed

*** 4. What is the age of this dog?**

- Less than 1 year
- 1-3 years
- 4-6 years
- Greater than 6 years

4. Description of Household and Dog Continued

* 5. What is the breed of this dog?

- ◯ Basset
- ◯ Beagle
- ◯ Bichon frise
- ◯ Boxer
- ◯ Bulldog
- ◯ Chihuahua
- ◯ Cocker spaniel
- ◯ Dachshund
- ◯ Doberman pinscher
- ◯ English springer spaniel
- ◯ German shepherd
- ◯ German shorthair pointer
- ◯ Golden retriever
- ◯ Great dane
- ◯ Labrador retriever
- ◯ Maltese
- ◯ Miniature pinscher
- ◯ Other
- ◯ Pekinese
- ◯ Pomeranian
- ◯ Poodle-miniature
- ◯ Poodle-standard
- ◯ Poodle-toy
- ◯ Pug
- ◯ Rottweiller
- ◯ Shetland sheep dog
- ◯ Shih Tsu
- ◯ Siberian husky
- ◯

- ◯ Welsh corgi
- ◯ West highland white terrier
- ◯ Yorkshire terrier

Other OR mixed breed (please specify)

5. Description of Household and Dog Continued

* 6. How well is this dog house trained?

- ◯ As an adult, almost never soils the house
- ◯ Occasionally soils the house
- ◯ Frequently soils the house

Other (please specify)

* 7. Regarding house training, which of the answers best describes the ease of house training of this dog?

- ◯ Was difficult to house train, and as an adult is still not well trained
- ◯ Was difficult to house train, although the dog is now well house trained
- ◯ Was easy to house train and remains well house trained
- ◯ Was almost completely house trained from the start (very few "accidents")

Other (please specify)

6. Questions About Stool Eating

* 8. At what age did you first notice your dog eating dog stools?

- ◯ Less than 1 year of age
- ◯ Between 1 year and 3 years of age
- ◯ Between 4 years and 10 years of age
- ◯ Over 10 years of age
- ◯ Unsure at what age

7. Treatments Tried to Stop Stool Eating

9. Management or behavior-modification procedures you may have attempted. Select all that apply.

- [ ] Nothing
- [ ] Pick up all, or most, stools
- [ ] Laced stools with pepper (embedded) to make them aversive
- [ ] Yell or chase away from stool
- [ ] Reward the dog for "leaving it alone"
- [ ] Use a citronella spray collar when the dog starts to eat stools
- [ ] Use a sound emitting or electronic collar when the dog starts to eat stools

Other (please specify)

10. If you have tried any of the behavior-modification procedures above, please respond as to whether or not any helped and be more specific in comment box

- ( ) Seemed to cure the problem
- ( ) Seemed to help, but not cure the problem
- ( ) Seemed to help only at first but no lasting cure
- ( ) Did not help

Comment

8. Treatments Tried to Stop Stool Eating

*** 11. Which of the following commercial dog food additive treatments have you tried?**

- [ ] 21st Century Deterrence
- [ ] Coproban
- [ ] Deter
- [ ] Dis-Taste
- [ ] For-Bid
- [ ] Nasty Habit
- [ ] NaturVet Deterrent
- [ ] Potty Mouth
- [ ] S.E.P
- [ ] Stop (Solve) Stool Eating
- [ ] Stop Tablets
- [ ] The Dog Poop Diet
- [ ] Other (specify below)
- [ ] I have not tried any of these

Other (please specify)

12. If you have tried any of the commercial food additives above, please respond as to whether or not any helped and be more specific in the comment box

- ○ Seemed to cure the problem
- ○ Seemed to help, but not cure the problem
- ○ Seemed to help only at first but no lasting cure
- ○ Did not help

Comment

9. Treatments Tried to Stop Stool Eating

13. Which non-commercial food additives have you tried?

- [ ] Pineapple
- [ ] Pepper
- [ ] Other (please specify below)

Other (please specify)

14. If you have tried any of the non-commercial food additives above, please respond as to whether or not any helped and be more specific in the comment box

- ◯ Seemed to cure the problem
- ◯ Seemed to help, but not cure the problem
- ◯ Seemed to help only at first but no lasting cure
- ◯ Did not help

Comment

10. Other animals' stools

11. Questions About Stool Eating

*** 15. What percent of dog stools that this dog has access to, does it usually eat?**

- ◯ Less than 25%
- ◯ 25-50%
- ◯ 50-75%
- ◯ 75-100%

12. Questions About Stool Eating

*** 16. How many times, total, have you seen your dog actually eat dog stools?**

- ◯ 1-5 times total
- ◯ 6-10 times total
- ◯ Greater than 10 times
- ◯ Never seen eat but I know that this dog eats stool

*** 17. Frequency of eating dog stools (best estimate)**

- ◯ 1 time per day or more
- ◯ Less than once a day, but at least 1 time per week
- ◯ Less than once a week, but at least 1 time per month
- ◯ Less than once a month, but at least 1 time per year
- ◯ Less than once a year

13. Questions About Stool Eating

*** 18. Main times of the day eating dog stools occurs, assuming stools are available (may be more than one time). Select all that apply.**

- ☐ Morning when just let (or goes) outside
- ☐ Morning after being fed
- ☐ Afternoon
- ☐ Late afternoon/evening before being fed
- ☐ Late afternoon/evening after being fed
- ☐ No particular main time

Comments:

*** 19. Locations where eating dog stools occurs? Select all that apply.**

- ☐ In yard
- ☐ In house
- ☐ On walks
- ☐ In off-leash dog parks
- ☐ In dog run or kennel anytime
- ☐ In dog run or kennel but only when alone for 2 or more hours
- ☐ In house, but when I am away
- ☐ In yard, but only when I am away

14. Questions About Stool Eating

20. Main times of the day, as listed above, that you think the dog is most bored

- ☐ Morning when just let (or goes) outside
- ☐ Morning after being fed
- ☐ Afternoon
- ☐ Late afternoon/evening before being fed
- ☐ Late afternoon/evening after being fed
- ☐ No particular main time

Comments:

* 21. If the dog only seems to eat dog stools when you are not around, how do you know (suspect) the dog ate a stool?

- ☐ Stool was there when I left but missing when I returned
- ☐ Stools are partially eaten
- ☐ The dog was alone so long, say 8 hours, that a stool would have been evident, but it was not
- ☐ Tell-tale breath odor
- ☐ Other way (specify below)

Other (please specify)

15. Questions About Stool Eating

22. Do you feel that boredom plays a role in your dog's stool eating?

- ◯ Yes, this is the main (or only) factor
- ◯ Maybe a partial reason but not the only reason
- ◯ Boredom plays no role in stool eating

Other (please specify)

16. Description of Household and Dog Continued

*** 23. What percentage of this dog's diet is of the following? The numbers must add up to 100.**

Kibble (dry)

Canned or semi-moist food

Raw food

People food

24. Please list the main types of food your pet currently eats, including the brand name when applicable, and the percentage of his/her diet each type of food contributes.

Kibble (dry)

Canned or semi-moist food

Raw food

People food

*** 25. What response below best describes your dog's eating behavior**

- Finicky eater
- Greedy eater, "wolfs down the food"
- Normal eater, neither finicky nor greedy

Other (please specify)

17. Description of Household and Dog Continued

26. How often do you take this dog on walks

- No one takes the dog on walks
- I do not take the dog on walks, but others do
- I take the dog on walks less than once a day, more than once a week
- I take the dog on walks 1-2 times per day
- I take the dog on walks 3-5 times per day

Other (please specify)

18. Description of Household and Dog Continued

*** 27. Where does your dog have access to dog stools?**

- [ ] In yard
- [ ] In house
- [ ] On walks
- [ ] In off-leash dog parks
- [ ] In dog run or kennel
- [ ] Other (please specify)

Other (please specify)

19. Description of Household and Dog Continued

*** 28. What problem behaviors, other than eating dog stools, does your dog seem to have? Select all that apply.**

- [ ] Separation anxiety
- [ ] Aggression to family members
- [ ] Aggression to other adults
- [ ] Aggression to other dogs in family
- [ ] Aggression to other non-family dogs
- [ ] Destructive behavior
- [ ] Excessive excitement
- [ ] Tail chasing or other compulsive-like behavior
- [ ] Excessive barking
- [ ] None of the above

Other (please specify)

20. Description of Household and Dog Continued

*** 29. Regarding obedience-type training, which response best fits this dog?**

- ◯ Had obedience training and is still well trained
- ◯ Had obedience training but is not now well trained
- ◯ No obedience training but is well trained
- ◯ No obedience training and is not well trained

Other (please specify)

*** 30. If you attempted some type of obedience training, which response best fits?**

- ◯ Was easily trained
- ◯ Was fairly easily trained
- ◯ Was difficult to train

Other (please specify)

21. Description of Household and Dog Continued

*** 31. What response below best describes the degree to which this dog is affectionate?**

	Very affectionate	Moderately affectionate	Relatively non-affectionate
Affection	◯	◯	◯

Other (please specify)

22. Description of Household and Dog Continued

*** 32. At what age did you adopt this dog?**

- ◯ Less than 2 months
- ◯ 2 to 4 months of age
- ◯ 4 to 6 months of age
- ◯ 6 months to 1 year of age
- ◯ 1-3 years of age
- ◯ 4-6 years of age
- ◯ Over 6 years of age

* 33. What do you know about your dogs mothering?

- ◯ Orphaned from mother and litter mates before 2 weeks of age
- ◯ Away from mother and litter mates after 2 weeks but before 7 weeks of age
- ◯ With mother for greater than seven weeks
- ◯ I do not know

Other (please specify)

23. Questions About Stool Eating

For the dog that at least occasionally eats stools, and which you described above, please answer the questions in this section about the stool eating

* 34. What types of dog stools does this dog eat?

- ◯ Only eats stools of other dogs
- ◯ Only eats its own stool
- ◯ Eats either its own or other dog's stools, whichever is available

Other (please specify)

* 35. Please indicate, to the best of your knowledge, the age of the dog stools that your dog eats. Select all that apply.

- ☐ Fresh stools, no more than 1 day old
- ☐ Stools 1 to 2 days old
- ☐ Stools 2 to 4 days old
- ☐ Stools older than 4 days old
- ☐ I do not know

Other (please specify)

24. Treatments Tried to Stop Stool Eating

This section deals with sources you have consulted to resolve the problem, the management or behavior-modification procedures you may have tried, and your possible attempts to solve the problem with a commercially available food additive

36. Please list the places you have consulted in an attempt to solve the problem. Select all that apply.

- [ ] Your veterinarian
- [ ] Friends who have dogs
- [ ] Dog trainers
- [ ] Behavior specialist who is certified (list type of certification, if known, below)
- [ ] Dog books (please name if covered by book)
- [ ] Online sources (list if discussed)
- [ ] Other

Please specify

25. Other

* **37. Dogs often eat the stools of other animals. Please select yes or no as to whether this dog has eaten stools of other animals.**

	Yes	No
Cat	○	○
Cow	○	○
Horse	○	○
Domestic or pet birds (chickens, parrots)	○	○
Wild birds (geese, ducks, crows)	○	○
Pigs	○	○
Sheep	○	○
Goats	○	○
If never has eaten feces of another animal, click "yes"	○	○

Other (please specify)

26. A Final Word

As you finish this survey, we want to sincerely thank you for taking the time to help us, as clinical animal behaviorists, to prevent and treat one of the disturbing and perplexing problem behaviors of companion dogs. We have one final question.

Why Dogs Eat Their Stools

38. Briefly provide any explanation you may have as to why some dogs engage in stool eating behavior and what are the best ways to prevent and solve the problem
