# Supplementary material for: The paradox of canine conspecific coprophagy
Source: Vet Med Sci. 2018 Jan 12;4(2):106–14. doi: 10.1002/vms3.92 (PMC5980124; doi:10.1002/vms3.92)
Supplement: Supplementary file 3 — Appendix S3. Responses to survey, dog behaviour. [file VMS3-4-106-s003.docx]

Appendix 3. Responses to survey, Dog Behavior: The Rest of the Story.
There were 1552 useable responses. However, not all respondents answered every question. For questions where "other" was listed as an option, the responses listed as “other” were deleted. The number of qualified responses for each question is indicated. Stool eaters are designated as having been seen eating dog stools 6 or more times and non-stool eaters never having been seen eating dog stools, leaving out dogs eating stools 1-5 times.

|  | | | | |  |
| --- | --- | --- | --- | --- | --- |
| **Number of dogs in your household** | | | | |  |
|  | | **All Dogs** | **Stool Eaters** | **Non-Stool Eaters** |  |
|  | | **Response Count**  **(%)** | **Response Count**  **(%)** | **Response Count**  **(%)** |  |
| One | | 489 (31.5%) | 45 (19.6%) | 371 (33.5%) |  |
| Two | | 510 (32.9%) | 72 (31.3%) | 377 (34.0%) |  |
| Three | | 244 (15.7%) | 53 (23.%) | 157 (14.2%) |  |
| Four | | 105 (6.8%) | 14 (6.1%) | 72 (6.5%) |  |
| More than four | | 204 (13.1%) | 46 (20.0%) | 131 (11.8%) |  |
| Qualified responses | | 1552 | 230 | 1108 |  |
|  | |  |  |  |  |
| **Outdoor Yard Space Available** | | | | |  |
|  | | **All Dogs** | **Stool Eaters** | **Non-Stool Eaters** |  |
|  | | **Response Count** | **Response Percent** | **Response Percent** |  |
| < 100 sq ft | | 144 (9.3%) | 12 (5.2%) | 108 (9.7%) |  |
| 100-500 sq ft | | 266 (17.1%) | 35 (15.2%) | 200 (18.1%) |  |
| 500-2000 sq ft | | 412 (26.5%) | 63 (27.4%) | 299 (27.0%) |  |
| 2000-5000 sq ft | | 349 (22.5%) | 62 (27.0%) | 244 (22.0%) |  |
| > 5000 sq ft | | 381 (24.5%) | 58 (25.2%) | 257 (23.2%) |  |
| Qualified responses | | 1552 | 230 | 1108 |  |
|  | |  |  |  |  |
| **Access to Yard Space** | | | | |  |
|  | | **All Dogs** | **Stool Eaters** | **Non-Stool Eaters** |  |
|  | | **Response Count** | **Response Percent** | **Response Percent** |  |
| Just the one dog I have | | 474 (30.5%) | 42 (18.3%) | 362 (32.7%) |  |
| I have multiple dogs and all have access to the space | | 1025 (66.%) | 173 (75.2%) | 715 (64.5%) |  |
| I have multiple dogs and all have access to the space | | 53 (3.4%) | 15 (6.5%) | 31 (2.8%) |  |
| Qualified responses | | 1552 | 230 | 1108 |  |
|  | |  |  |  |  |
| **Access to Grass** | | | | |  |
|  | |  | **Stool Eaters** | **Non-Stool Eaters** |  |
|  | | **Response Count** | **Response Percent** | **Response Percent** |  |
| Yes | | 1424 (91.8%) | 210 (91.3%) | 1020 (92.1%) |  |
| No | | 128 (8.2%) | 20 (8.7%) | 88 (7.9%) |  |
| Qualified responses | | 1552 | 230 | 1108 |  |
|  | |  |  |  |  |
| **Gender-neuter category** | | | | |  |
|  | | **All Dogs** | **Stool Eaters** | **Non-Stool Eaters** |  |
|  | | **Response Count** | **Response Percent** | **Response Percent** |  |
| Male-intact | | 146 (9.4%) | 14 (6.1%) | 113 (10.2%) |  |
| Male-neuter | | 643 (41.4%) | 104 (45.2%) | 458 (41.3%) |  |
| Female-intact | | 123 (7.9%) | 16 (7.0%) | 86 (7.8%) |  |
| Female-spayed | | 640 (41.2%) | 96 (41.7%) | 451 (40.7%) |  |
| Qualified responses | | 1552 | 230 | 1108 |  |
|  | |  |  |  |  |
| **Age** | | | | |  |
|  | | **All Dogs** | **Stool Eaters** | **Non-Stool Eaters** |  |
|  | | **Response Count** | **Response Percent** | **Response Percent** |  |
| Less than 1 year | | 48 (3.1%) | 4 (1.7%) | 35 (3.2%) |  |
| 1-3 years | | 419 (27.2%) | 53 (23.1%) | 300 (27.2%) |  |
| 4-6 years | | 379 (24.6%) | 66 (28.8%) | 265 (24.0%) |  |
| Greather than 6 years | | 701 (45.4%) | 106 (46.3%) | 504 (45.7%) |  |
|  | |  |  |  |  |
| Qualified responses | | 1543 | 229 | 1104 |  |
|  | |  |  |  |  |
| **Age at Adoption** | | | | |  |
|  | | **All Dogs** | **Stool Eaters** | **Non-Stool Eaters** |  |
|  | | **Response Count** | **Response Percent** | **Response Percent** |  |
| Less than 2 months | | 330 (21.3%) | 55 (24.0%) | 235 (21.3%) |  |
| 2 to 4 months of age | | 643 (41.6%) | 98 (42.8%) | 451 (40.8%) |  |
| 4 to 6 months of age | | 93 (6.0%) | 13 (5.7%) | 64 (5.8%) |  |
| 6 months to 1 year of age | | 169 (10.9%) | 23 (10.0%) | 120 (10.9%) |  |
| 1-3 years of age | | 227 (14.7%) | 31 (13.5%) | 172 (15.6%) |  |
| 4-6 years of age | | 54 (3.5%) | 5 (2.2%) | 42 (3.8%) |  |
| Over 6 years of age | | 30 (1.9%) | 4 (1.7%) | 21 (1.9%) |  |
|  | |  |  |  |  |
| Qualified responses | | 1546 | 229 | 1105 |  |
|  | |  |  |  |  |
| **Mothering** | | | | |  |
|  | | **All Dogs** | **Stool Eaters** | **Non-Stool Eaters** |  |
|  | | **Response Count** | **Response Percent** | **Response Percent** |  |
| Orphaned from mother and litter mates before 2 weeks of age | | 15 (1.6%) | 1 (0.6%) | 14 (1.3%) |  |
| Away from mother and litter mates after 2 weeks but before 7 weeks | | 156 (16.5%) | 21 (13.4%) | 112 (10.5%) |  |
| With mother for greater than seven weeks | | 775 (51.7%) | 135 (59.1%) | 530 (49.7%) |  |
|  | |  |  |  |  |
| Qualified responses | | 1449 | 227 | 1067 |  |
|  | |  |  |  |  |
| **Main Diet** | | | | |  |
|  | | **All Dogs** | **Stool Eaters** | **Non-Stool Eaters** |  |
|  | | **Response Count** | **Response Percent** | **Response Percent** |  |
| Kibble (dry) | | 1109 (79.4%) | 172 (82.3%) | 782 (78.3%) |  |
| Canned or semi-moist food | | 48 (3.4%) | 1 (0.5%) | 38 (3.8%) |  |
| Raw food | | 223 (16.0%) | 33 (15.8%) | 168 (16.8%) |  |
| People food | | 16 (1.1%) | 3 (1.4%) | 11 (1.1%) |  |
|  | |  |  |  |  |
| Qualified responses | | 1396 | 209 | 999 |  |
|  | |  |  |  |  |
| **Breed** | | | | |  |
|  | | **All Dogs** | **Stool Eaters** | **Non-Stool Eaters** |  |
|  | | **Response Count** | **Response Percent** | **Response Percent** |  |
| Australian shepherd | | 45 (2.9%) | 9 (3.9%) | 30 (2.7%) |  |
| Basset | | 11 (0.7%) | 4 (1.7%) | 6 (.5%) |  |
| Beagle | | 20 (1.3%) | 6 (2.6%) | 10 (0.9%) |  |
| Bichon frise | | 6 (0.4%) | 0 (0%) | 5 (0.5%) |  |
| Boxer | | 18 (1.2%) | 1 (0.4%) | 13 (1.2%) |  |
| Bulldog | | 2 (0.1%) | 0 (0%) | 2 (0.2%) |  |
| Chihuahua | | 21 (1.4%) | 4 (1.7%) | 13 (1.2%) |  |
| Cocker spaniel | | 11 (0.7%) | 2 (0.9%) | 8 (0.7%) |  |
| Dachshund | | 21 (1.4%) | 4 (1.7%) | 10 (0.9%) |  |
| Doberman pinscher | | 8 (0.5%) | 1 (0.4%) | 7 (0.6%) |  |
| English springer | | 8 (0.5%) | 0 (0%) | 7 (0.6%) |  |
| German shepherd | | 88 (5.7%) | 15 (6.5%) | 57 (5.1%) |  |
| German shorthair | | 12 (0.8%) | 2 (0.9%) | 9 (0.8%) |  |
| Golden retriever | | 59 (3.8%) | 10 (4.3%) | 43 (3.9%) |  |
| Great dane | | 12 (0.8%) | 2 (0.9%) | 8 (0.7%) |  |
| Jack Russell terrier | | 19 (1.2%) | 5 (2.2%) | 12 (1.1%) |  |
| Labrador retriever | | 60 (3.9%) | 9 (3.9%) | 44 (4.0%) |  |
| Maltese | | 4 (0.3%) | 0 (0%) | 4 (0.4%) |  |
| Miniature pinscher | | 6 (0.4%) | 0 (0%) | 3 (0.3%) |  |
| Pekinese | | 3 (0.2%) | 0 (0%) | 1 (0.1%) |  |
| Pomeranian | | 12 (0.8%) | 1 (0.4%) | 6 (0.5%) |  |
| Poodle-miniature | | 9 (0.6%) | 0 (0%) | 6 (0.5%) |  |
| Poodle-standard | | 19 (1.2%) | 0 (0%) | 16 (1.4%) |  |
| Poodle-toy | | 8 (0.5%) | 0 (0%) | 7 (0.6%) |  |
| Pug | | 12 (0.8%) | 1 (0.4%) | 9 (0.8%) |  |
| Rottweiler | | 16 (1.0%) | 1 (0.4%) | 13 (1.2%) |  |
| Shetland sheep dog | | 30 (1.9%) | 11 (4.8%) | 16 (1.4%) |  |
| Shih Tsu | | 10 (0.6%) | 1 (0.4%) | 7 (0.6%) |  |
| Siberian husky | | 27 (1.7%) | 4 (1.7%) | 18 (1.6%) |  |
| Welsh corgi | | 13 (0.8%) | 4 (1.7%) | 6 (0.5%) |  |
| West highland white terrier | | 4 (0.3%) | 3 (1.3%) | 1 (0.1%) |  |
| Yorkshire terrier | | 9 (0.6%) | 2 (0.9%) | 6 (0.5%) |  |
| Mixed breed | | 314 (20.2%) | 44 (19.1%) | 242 (21.8%) |  |
| Other | | 635 (40.9%) | 84 (36.5%) | 463 (41.8%) |  |
| Answered Question | | 1552 | 230 | 1108 |  |
|  | |  |  |  |  |
|  | |  |  |  |  |
| **Eating Behavior** | | | | |  |
|  | | **All Dogs** | **Stool Eaters** | **Non-Stool Eaters** |  |
|  | | **Response Count** | **Response Percent** | **Response Percent** |  |
| Finicky eater | | 155 (10.6%) | 7 (3.1%) | 124 (11.8%) |  |
| Greedy eater | | 465 (31.7%) | 114 (51.1%) | 297 (28.2%) |  |
| Normal eater | | 845 (57.7%) | 102 (45.7%) | 633 (60.1%) |  |
|  | |  |  |  |  |
| Qualified responses | | 1465 | 223 | 1054 |  |
|  | |  |  |  |  |
| **Affection Level** | | | | |  |
|  | | **All Dogs** | **Stool Eaters** | **Non-Stool Eaters** |  |
|  | | **Response Count** | **Response Percent** | **Response Percent** |  |
| Relatively non-affectionate | | 62 (4.1%) | 13 (5.8%) | 39 (3.6%) |  |
| Moderately affectionate | | 476 (31.8%) | 74 (33.0%) | 343 (31.8%) |  |
| Very affectionate | | 958 (64.0%) | 137 (61.2%) | 698 (64.6%) |  |
|  | |  |  |  |  |
| Qualified responses | | 1496 | 224 | 1080 |  |
|  | |  |  |  |  |
| **Ease of House Training** | | | | |  |
|  | | **All Dogs** | **Stool Eaters** | **Non-Stool Eaters** |  |
|  | | **Response Count** | **Response Percent** | **Response Percent** |  |
| Was difficult to house train, and as an adult is still not well trained | | 55 (4.0%) | 9 (4.3%) | 37 (3.8%) |  |
| Was difficult to house train, although the dog is now well house trained | | 182 (13.3%) | 35 (16.7%) | 120 (12.2%) |  |
| Was easy to house train and remains well house trained | | 1106 (80.8%) | 163 (78.0%) | 804 (82.0%) |  |
| Was easy to house train, but as an adult is not well house trained | | 25 (1.8%) | 2 (1.0%) | 20 (2.0%) |  |
|  | |  |  |  |  |
| Qualified responses | | 1368 | 209 | 981 |  |
|  | |  |  |  |  |
| **Problem Behaviors** | | | | |  |
|  | | **All Dogs** | **Stool Eaters** | **Non-Stool Eaters** |  |
| **Answer Options** | | **Response Count** | **Response Percent** | **Response Percent** |  |
| This dog does not have any problem behaviors | | 560 (36.5%) | 75 (32.6%) | 407 (36.7%) |  |
| Separation anxiety | | 239 (15.6%) | 33 (14.3%) | 174 (15.7%) |  |
| Aggression to family members | | 15 (1.0%) | 1 (0.4%) | 13 (1.2%) |  |
| Aggression to other adults | | 54 (3.5%) | 6 (2.6%) | 41 (3.7%) |  |
| Aggression to other dogs in family | | 56 (3.6%) | 9 (3.9%) | 41 (3.7%) |  |
| Aggression to other non-family dogs | | 238 (15.5%) | 33 (14.3%) | 181 (16.3%) |  |
| Destructive behavior | | 66 (4.3%) | 13 (5.7%) | 42 (3.8%) |  |
| Excessive excitability | | 231 (15.0%) | 45 (19.6%) | 154 (13.9%) |  |
| Tail chasing or other compulsive-like behavior | | 46 (3.0%) | 8 (3.5%) | 32 (2.9%) |  |
| Excessive barking | | 147 (9.6%) | 29 (12.6%) | 96 (8.7%) |  |
| Urinating or defecating in the house | | 81 (5.3%) | 15 (6.5%) | 55 (5.0%) |  |
| Other | | 384 (25.0%) | 63 (27.4%) | 273 (24.6%) |  |
|  | | 1535 | 230 | 1108 |  |
|  | |  |  |  |  |
|  | |  |  |  |  |
|  | |  |  |  |  |
| **Stool Eating** | | | | |  |
|  | | **All Dogs** | **Stool Eaters** | **Non-Stool Eaters** |  |
|  | | **Response Count** | **Response Percent** | **Response Percent** |  |
| None that I know about | | 1108 (76.9%) | 0 (0%) | 1108 (100.%) |  |
| 1-5 times total | | 103 (7.1%) | 0 (0%) | 0 (0%) |  |
| 6-10 times total | | 47 (3.3%) | 47 (20.4%) | 0 (0%) |  |
| Greater than 10 times | | 183 (12.7%) | 183 (79.6%) | 0 (0%) |  |
|  | |  |  |  |  |
| Qualified responses | | 1441 | 230 | 1108 |  |
|  | |  |  |  |  |
|  |  |  |  |  |  |
| **Type of Stool Eaten For Stool Eating Dogs Only** | | | | |  |
|  | |  | **Stool Eaters** |  |  |
|  | |  | **Response Percent** |  |  |
|  | |  |  |  |  |
| Only eats stools of other dogs | |  | 72 (37.9%) |  |  |
| Only eats its own stool | |  | 35 (18.4%) |  |  |
| Eats either its own or other dog's stools, whichever is available | |  | 83 (43.7%) |  |  |
|  | |  |  |  |  |
|  | |  |  |  |  |
| Qualified responses | |  | 190 |  |  |
|  | |  |  |  |  |
| **Frequency of Stool Eating. For Stool Eaters Only** | | | | |  |
|  | |  | **Stool Eaters** |  |  |
|  | |  | **Response Percent** |  |  |
|  | |  |  |  |  |
| 1 time per day or more | |  | 47 (30.3%) |  |  |
| Less than once a day, but at least 1 time per week | |  | 40 (25.8%) |  |  |
| Less than once a week, but at least 1 time per month | |  | 37 (23.9%) |  |  |
| Less than once a month, but at least 1 time per year | |  | 31 (20.0%) |  |  |
|  | |  |  |  |  |
| Qualified responses | |  | 155 |  |  |
| **Age of Stool of Stool Eaten for Stool Eaters** | | | | |  |
|  | |  | **Stool Eaters** |  |  |
|  | |  | **Response Percent** |  |  |
|  | |  |  |  |  |
| Fresh stools, up to 2 days old | |  | 157 (81.3%) |  |  |
| Stools 2 to 4 days old | |  | 12 (6.2%) |  |  |
| Stools older than 4 days old | |  | 4 (2.1%) |  |  |
| Stools of any age | |  | 20 (10.4%) |  |  |
| Qualified responses | |  | 193 |  |  |
|  | |  |  |  |  |
| **Eating Grass** | | | | |  |
|  | | **All Dogs** | **Stool Eaters** | **Non-Stool Eaters** |  |
|  | | **Response Count** | **Response Percent** | **Response Percent** |  |
| Never | | 122 (8.2%) | 14 (6.1%) | 94 (8.5%) |  |
| At least once but less than 6 times | | 263 (17.6%) | 40 (17.4%) | 200 (18.1%) |  |
| 6 to 10 times | | 148 (9.9%) | 25 (10.9%) | 103 (9.3%) |  |
| More than 10 times | | 963 (64.4%) | 151 (65.7%) | 711 (64.2%) |  |
| Answered Question | | 1496 | 230 | 1108 |  |
|  | |  |  |  |  |
