# Supplementary material for: The paradox of canine conspecific coprophagy
Source: Vet Med Sci. 2018 Jan 12;4(2):106–14. doi: 10.1002/vms3.92 (PMC5980124; doi:10.1002/vms3.92)
Supplement: Supplementary file 4 — Appendix S4. Responses to why dogs eat their stools. [file VMS3-4-106-s004.doc]

Appendix 4. Responses to Why Dogs Eat Their Stools. There were 2,561 total with 1,475 for returns for dogs being seen eating stools greater than 10 times and at least on a weekly basis. See text for details. In calculating the percent of returns for some variables, the percent calculation excluded those responses giving of “I don’t know” or “other.”

**#1: Number of dogs in the household**

|  | # of dogs | % of total |
| --- | --- | --- |
| One | 94 | 6.4% |
| Two | 339 | 23.0% |
| Three | 303 | 20.5% |
| Four | 215 | 14.6% |
| More than four | 524 | 35.5% |

**#2: Dogs seen eating dog stools in multi-dog household**

|  | # of dogs | % of total |
| --- | --- | --- |
| Only one | 644 | 43.7% |
| Two | 407 | 27.6% |
| Three | 189 | 12.8% |
| Four | 81 | 5.5% |
| More than 4 | 154 | 10.4% |
| None | 0 | 0.0% |
| None...they only eat cat, horse, or other animal's stools |  | 0.0% |

**#3: Sex and neuter status of the stool-eating dog**

|  | # of dogs | % of total |
| --- | --- | --- |
| Male-intact | 132 | 8.9% |
| Male-neutered | 443 | 30.0% |
| Female-intact | 285 | 19.3% |
| Female-spayed | 615 | 41.7% |

**#4: Age of dog**

|  | # of dogs | % of total |
| --- | --- | --- |
| Less than 1 year | 108 | 7.3% |
| 1-3 years | 418 | 28.3% |
| 4-6 years | 336 | 22.8% |
| More than 6 years | 613 | 41.6% |

**#5: Breed of the dog**

|  | # of dogs | % of total |
| --- | --- | --- |
| Shih Tsu | 15 | 1.0% |
| Poodle-standard | 2 | 0.1% |
| Poodle-miniature | 5 | 0.3% |
| Poodle-toy | 2 | 0.1% |
| West highland white terrier | 5 | 0.3% |
| English springer spaniel | 12 | 0.8% |
| Boxer | 6 | 0.4% |
| Doberman pinscher | 4 | 0.3% |
| Shetland sheep dog | 70 | 4.7% |
| Labrador retriever | 153 | 10.4% |
| Cocker spaniel | 22 | 1.5% |
| Golden retriever | 85 | 5.8% |
| Great dane | 21 | 1.4% |
| Pekinese | 1 | 0.1% |
| Yorkshire terrier | 16 | 1.1% |
| Dachshund | 46 | 3.1% |
| Siberian husky | 5 | 0.3% |
| Basset | 81 | 5.5% |
| Rottweiller | 12 | 0.8% |
| German shepherd | 78 | 5.3% |
| Beagle | 46 | 3.1% |
| Pomeranian | 24 | 1.6% |
| Miniature pinscher | 8 | 0.5% |
| Bichon frise | 1 | 0.1% |
| German shorthair pointer | 13 | 0.9% |
| Bulldog | 15 | 1.0% |
| Maltese | 2 | 0.1% |
| Welsh corgi | 43 | 2.9% |
| Chihuahua | 16 | 1.1% |
| Pug | 3 | 0.2% |
| Other | 663 | 44.9% |

**#6: How well dog is house trained**

|  | # of dogs | % of total |
| --- | --- | --- |
| As an adult, almost never soils the house | 1207 | 81.8% |
| Occasionally soils the house | 220 | 14.9% |
| Frequently soils the house | 48 | 3.3% |

**#7: Ease of house training the dog**

|  | # of dogs | % of total |
| --- | --- | --- |
| Difficult to house train, and as an adult is still not well trained | 156 | 10.6% |
| Difficult to house train, although the dog is now well house trained | 221 | 15.0% |
| Easy to house train and remains well house trained | 707 | 47.9% |
| Almost completely house trained from the start | 391 | 26.5% |

**#8: Age dog first started eating dog stools**

|  | # of dogs | % of total |
| --- | --- | --- |
| Less than 1 year of age | 692 | 46.9% |
| Between 1 year and 3 years of age | 466 | 31.6% |
| Between 4 years and 10 years of age | 197 | 13.4% |
| Over 10 years of age | 29 | 2.0% |
| Unsure at what age | 91 | 6.2% |

**#9: Management or behavior-modification procedures attempted**

|  | # | % of total |
| --- | --- | --- |
| Nothing | 87 | 2.4% |
| Pick up all, or most, stools | 1323 | 36.4% |
| Laced stools with pepper (embedded) to make them aversive | 295 | 8.1% |
| Yell or chase away from stool | 1048 | 28.8% |
| Reward the dog for "leaving it alone" | 424 | 11.7% |
| Use a citronella spray collar when the dog starts to eat stools | 27 | 0.7% |
| Use a sound emitting or electronic collar when the dog starts to eat stools | 56 | 1.5% |
| Other (please specify) | 379 | 10.4% |

**#10: Success of management or behavior-modification procedures attempted**

|  | Seemed to cure the problem | Seemed to help, but not cure the problem | Seemed to help only at first but no lasting cure | Did not help |
| --- | --- | --- | --- | --- |
| Nothing | 1 | 8 | 10 | 40 |
| Pick up all, or most, stools | 24 | 355 | 276 | 594 |
| Laced stools with pepper (embedded) to make them aversive | 3 | 48 | 83 | 155 |
| Yell or chase away from stool | 11 | 235 | 236 | 536 |
| Reward the dog for "leaving it alone" | 15 | 127 | 106 | 173 |
| Use a citronella spray collar when the dog starts to eat stools | 3 | 1 | 12 | 11 |
| Use a sound emitting or electronic collar when the dog starts to eat stools | 1 | 11 | 21 | 23 |
| Other (please specify) | 15 | 98 | 89 | 153 |

**#11: Commercial dog food additive treatments tried**

|  | # tried | % of total |
| --- | --- | --- |
| 21st Century Deterrence | 6 | 0.3% |
| Coproban | 58 | 3.0% |
| Deter | 238 | 12.1% |
| Dis-Taste | 154 | 7.9% |
| For-Bid | 352 | 18.0% |
| Nasty Habit | 13 | 0.7% |
| NaturVet Deterrent | 20 | 1.0% |
| Potty Mouth | 24 | 1.2% |
| S.E.P | 58 | 3.0% |
| Stop (Solve) Stool Eating | 27 | 1.4% |
| Stop Tablets | 26 | 1.3% |
| The Dog Poop Diet | 6 | 0.3% |
| Other (specify below) | 102 | 5.2% |
| I have not tried any of these | 875 | 44.7% |

**#12: Success of commercial dog food additive treatments tried**

|  | Seemed to cure the problem | Seemed to help, but not cure the problem | Seemed to help only at first but no lasting cure | Did not help |
| --- | --- | --- | --- | --- |
| 21st Century Deterrence | 0 | 0 | 3 | 3 |
| Coproban | 1 | 5 | 14 | 37 |
| Deter | 2 | 19 | 52 | 162 |
| Dis-Taste | 1 | 15 | 27 | 111 |
| For-Bid | 3 | 32 | 77 | 234 |
| Nasty Habit | 0 | 1 | 6 | 6 |
| NaturVet Deterrent | 0 | 2 | 3 | 13 |
| Potty Mouth | 0 | 6 | 6 | 11 |
| S.E.P | 0 | 4 | 14 | 39 |
| Stop (Solve) Stool Eating | 0 | 2 | 7 | 18 |
| Stop Tablets | 0 | 2 | 3 | 20 |
| The Dog Poop Diet | 0 | 0 | 1 | 5 |
| Other (specify below) | 3 | 13 | 19 | 50 |
| I have not tried any of these | 1 | 3 | 5 | 73 |

**#13: Non-commercial food additives tried**

|  | # | % of total |
| --- | --- | --- |
| Pineapple | 364 | 27.4% |
| Pepper | 181 | 13.6% |
| Other (please specify below) | 335 | 25.2% |
| Other (please specify) | 448 | 33.7% |

**#14: Success of non-commercial food additives tried**

|  | Seemed to cure the problem | Seemed to help, but not cure the problem | Seemed to help only at first but no lasting cure | Did not help |
| --- | --- | --- | --- | --- |
| Pineapple | 6 | 48 | 56 | 248 |
| Pepper | 1 | 10 | 33 | 136 |
| Other (please specify below) | 9 | 38 | 54 | 179 |

**#15: Percent of dog stools that dog has access to and usually eats**

|  | # of dogs | % of total |
| --- | --- | --- |
| < 25% | 543 | 36.8% |
| 25-50% | 387 | 26.2% |
| 50-75% | 244 | 16.5% |
| 75-100% | 301 | 20.4% |

**#16: Total number of times dog was seen eating stool**

|  | # of dogs | % of total |
| --- | --- | --- |
| Greater than 10 times | 1475 | 100% |

**#17: Frequency of eating dog stools**

|  | # of dogs | % of total |
| --- | --- | --- |
| Daily | 916 | 62.1% |
| Weekly | 559 | 37.9% |

**#18: Main times of the day eating dog stools occur, assuming stools are available**

|  | # | % of total |
| --- | --- | --- |
| Morning when just let (or goes) outside | 153 | 8.2% |
| Morning after being fed | 174 | 9.3% |
| Afternoon | 111 | 5.9% |
| Late afternoon/evening before being fed | 111 | 5.9% |
| Late afternoon/evening after being fed | 203 | 10.8% |
| No particular main time | 1122 | 59.9% |

**#19: Locations where eating dog stools occurs**

|  | # | % of total |
| --- | --- | --- |
| In yard | 1329 | 64.3% |
| In house | 191 | 9.2% |
| On walks | 183 | 8.8% |
| In off-leash dog parks | 94 | 4.5% |
| In dog run or kennel anytime | 169 | 8.2% |
| In dog run or kennel but only when alone for 2 or more hours | 9 | 0.4% |
| In house, but when I am away | 34 | 1.6% |
| In yard, but only when I am away | 59 | 2.9% |

**#20: Main times of the day that dog is likely most bored**

|  | # | % of total |
| --- | --- | --- |
| Morning when just let (or goes) outside | 21 | 1.4% |
| Morning after being fed | 66 | 4.3% |
| Afternoon | 223 | 14.6% |
| Late afternoon/evening before being fed | 88 | 5.8% |
| Late afternoon/evening after being fed | 60 | 3.9% |
| No particular main time | 1067 | 70.0% |

**#21: If dog only seems to eat dog stools when owner is not around, how does the owner know the dog ate a stool?**

|  | # | % of total |
| --- | --- | --- |
| Stool was there when I left but missing when I returned | 252 | 13.2% |
| Stools are partially eaten | 368 | 19.2% |
| The dog was alone so long, say 8 hours, that a stool would have been evident, but it was not | 27 | 1.4% |
| Tell-tale breath odor | 734 | 38.4% |
| Other way (specify below) | 531 | 27.8% |

**#22: Does boredom play a role in dog’s stool eating?**

|  | # | % of total |
| --- | --- | --- |
| Yes, this is the main (or only) factor | 9 | 0.5% |
| Maybe a partial reason but not the only reason | 293 | 17.8% |
| Boredom plays no role in stool eating | 1105 | 67.3% |
| Other (please specify) | 236 | 14.4% |

**#23: Dog’s diet**

|  | Mean % of diet |
| --- | --- |
| Kibble | 88.1% |
| Canned or semi-moist | 12.5% |
| Raw food | 27.90% |
| People food | 7.40% |

**#25: Dog’s eating behavior**

|  | # of dogs | % of total |
| --- | --- | --- |
| Finicky eater | 54 | 3.7% |
| Greedy eater, "wolfs down the food" | 757 | 52.2% |
| Normal eater, neither finicky nor greedy | 554 | 38.2% |
| Other (please specify) | 84 | 5.8% |

**#26: How often dog is taken on walks**

|  | # | % of total |
| --- | --- | --- |
| No one takes the dog on walks | 379 | 23.7% |
| I do not take the dog on walks, but others do | 26 | 1.6% |
| I take the dog on walks less than once a day, more than once a week | 406 | 25.4% |
| I take the dog on walks 1-2 times per day | 376 | 23.5% |
| I take the dog on walks 3-5 times per day | 75 | 4.7% |
| Other (please specify) | 335 | 21.0% |

**#27: Dog’s access to dog stools**

|  | # | % of total |
| --- | --- | --- |
| In yard | 1267 | 64.5% |
| In house | 137 | 7.0% |
| On walks | 243 | 12.4% |
| In off-leash dog parks | 122 | 6.2% |
| In dog run or kennel | 159 | 8.1% |
| Other (please specify) | 35 | 1.8% |

**#28: Other problem behaviors**

|  | # | % of total |
| --- | --- | --- |
| Separation anxiety | 134 | 6.9% |
| Aggression to family members | 10 | 0.5% |
| Aggression to other adults | 28 | 1.4% |
| Aggression to other dogs in family | 78 | 4.0% |
| Aggression to other non-family dogs | 128 | 6.6% |
| Destructive behavior | 71 | 3.7% |
| Excessive excitement | 209 | 10.8% |
| Tail chasing or other compulsive-like behavior | 36 | 1.9% |
| Excessive barking | 146 | 7.5% |
| None of the above | 849 | 43.9% |
| Other (please specify) | 246 | 12.7% |

**#29: Obedience training**

|  | # | % of total |
| --- | --- | --- |
| Had obedience training and is still well trained | 742 | 47.6% |
| Had obedience training but is not now well trained | 125 | 8.0% |
| No obedience training but is well trained | 403 | 25.9% |
| No obedience training and is not well trained | 85 | 5.5% |
| Other (please specify) | 203 | 13.0% |

**#30: Success of attempted obedience training**

|  | # | % of total |
| --- | --- | --- |
| Was easily trained | 665 | 44.6% |
| Was fairly easily trained | 546 | 36.6% |
| Was difficult to train | 144 | 9.7% |
| Other (please specify) | 136 | 9.1% |

**#31: Dog’s affection level**

|  | # of dogs | % of total |
| --- | --- | --- |
| Affection - Very affectionate | 999 | 70.5% |
| Affection - Moderately affectionate | 313 | 22.1% |
| Affection - Relatively non-affectionate | 40 | 2.8% |
| Other (please specify) | 66 | 4.7% |

**#32: Age at adoption**

|  | # | % of total |
| --- | --- | --- |
| Less than 2 months | 389 | 28.8% |
| 2 to 4 months of age | 441 | 32.7% |
| 4 to 6 months of age | 94 | 7.0% |
| 6 months to 1 year of age | 122 | 9.0% |
| 1-3 years of age | 181 | 13.4% |
| 4-6 years of age | 69 | 5.1% |
| Over 6 years of age | 53 | 3.9% |

**#33: Dog’s mothering**

|  | # | % of total |
| --- | --- | --- |
| Orphaned from mother and litter mates before 2 weeks of age | 10 | 0.6% |
| Away from mother and litter mates after 2 weeks but before 7 weeks of age | 96 | 6.1% |
| With mother for greater than seven weeks | 822 | 52.3% |
| I do not know | 421 | 26.8% |
| Other (please specify) | 224 | 14.2% |

**#34: Type of dog stools dog eats**

|  | # of dogs | % of total |
| --- | --- | --- |
| Only eats stools of other dogs | 475 | 32.0% |
| Only eats its own stool | 151 | 10.2% |
| Eats either its own or other dog's stools, whichever is available | 717 | 48.3% |
| Other (please specify) | 142 | 9.6% |

**#35: Age of dog stools that dog eats**

|  | # of dogs | % of total that know |
| --- | --- | --- |
| Fresh stools, no more than 1 day old | 702 | 57.1% |
| Stools 1 to 2 days old | 340 | 27.9% |
| Stools 2 to 4 days old | 99 | 8.1% |
| Stools older than 4 days old | 78 | 6.4% |
| I do not know | 120 |  |
| Other (please specify) | 136 |  |

**#36: Places consulted in an attempt to solve the problem**

|  | # | % of total |
| --- | --- | --- |
| Your veterinarian | 728 | 23.6% |
| Friends who have dogs | 827 | 26.8% |
| Dog trainers | 443 | 14.4% |
| Behavior specialist who is certified (list type of certification, if known, below) | 96 | 3.1% |
| Dog books (please name if covered by book) | 280 | 9.1% |
| Online sources (list if discussed) | 540 | 17.5% |
| Other | 173 | 5.6% |

**#37: Eating of other animals’ stools**

|  | # | % of subtotal | % of grand total (all dogs) |
| --- | --- | --- | --- |
| Cat - Yes | 555 | 49.4% | 6.3% |
| Cat - No | 569 | 50.6% | 6.5% |
| Subtotal | 1124 |  |  |
| Cow - Yes | 135 | 13.5% | 1.5% |
| Cow - No | 867 | 86.5% | 9.8% |
| Subtotal | 1002 |  |  |
| Horse - Yes | 324 | 31.0% | 3.7% |
| Horse - No | 721 | 69.0% | 8.2% |
| Subtotal | 1045 |  |  |
| Domestic or pet birds (chickens, parrots) - Yes | 102 | 10.6% | 1.2% |
| Domestic or pet birds (chickens, parrots) - No | 864 | 89.4% | 9.8% |
| Subtotal | 966 |  |  |
| Wild birds (geese, ducks, crows) - Yes | 269 | 26.9% | 3.1% |
| Wild birds (geese, ducks, crows) - No | 732 | 73.1% | 8.3% |
| Subtotal | 1001 |  |  |
| Pigs - Yes | 12 | 1.3% | 0.1% |
| Pigs - No | 922 | 98.7% | 10.5% |
| Subtotal | 934 |  |  |
| Sheep - Yes | 119 | 12.3% | 1.4% |
| Sheep - No | 848 | 87.7% | 9.6% |
| Subtotal | 967 |  |  |
| Goats - Yes | 60 | 6.4% | 0.7% |
| Goats - No | 877 | 93.6% | 10.0% |
| Subtotal | 937 |  |  |
| Has never eaten feces of another animal | 428 | 52.3% | 4.9% |
| Has eaten feces of another animal | 391 | 47.7% | 4.4% |
| Subtotal | 819 |  |  |
| Other (please specify) | 15 |  | 0.2% |
| Grand total (all dogs) | 8810 |  |  |
